# Development of a novel lateral flow assay for detection of African swine fever in blood

**DOI:** 10.1186/s12917-016-0831-4

**Published:** 2016-09-15

**Authors:** P. Sastre, C. Gallardo, A. Monedero, T. Ruiz, M. Arias, A. Sanz, P. Rueda

**Affiliations:** 1 Inmunología y Genética Aplicada S. A. (INGENASA), Madrid, Spain; 2 European Union Reference Laboratory for ASF (EURL), Centro de Investigación en Sanidad Animal, INIA, Madrid, Spain

**Keywords:** African swine fever virus, Lateral flow assay, Diagnosis

## Abstract

**Background:**

African swine fever (ASF) is a viral infectious disease of domestic and wild suids of all breeds and ages, causing a wide range of hemorrhagic syndromes and frequently characterized by high mortality. The disease is endemic in Sub-Saharan Africa and Sardinia. Since 2007, it has also been present in different countries of Eastern Europe, where control measures have not been effective so far. The continued spread poses a serious threat to the swine industry worldwide. In the absence of vaccine, early detection of infected animals is of paramount importance for control of the outbreak, to prevent the transmission of the virus to healthy animals and subsequent spreading of the disease. Current laboratory diagnosis is mainly based on virological methods (antigen and genome detection) and serodiagnosis.

**Results:**

In the present work, a Lateral Flow Assay (LFA) for antigen detection has been developed and evaluated. The test is based on the use of a MAb against VP72 protein of ASFV, the major viral capsid protein and highly immunogenic. First experiments using VP72 viral and recombinant protein or inactivated culture virus showed promising results with a sensitivity similar to that of a commercially available Antigen-ELISA. Moreover, these strips were tested with blood from experimentally infected pigs and field animals and the results compared with those of PCR and Antigen-ELISA. For the experimentally infected samples, there was an excellent correlation between the LFA and the ELISA, while the PCR always showed to be more sensitive (38 % positive samples by PCR versus 27 % by LFA). The LFA was demonstrated to be positive for animals with circulating virus levels exceeding 10^4^ HAU. With the field samples, once again, the PCR detected more positives than either the Antigen-ELISA or LFA, although here the number of positive samples scored by the LFA exceeded the values obtained with the Antigen-ELISA, showing 60 % positivity *vs* 48 % for the ELISA. For the two groups of sera, the specificity was close to 100 % indicating that hardly any false positive samples were found.

**Conclusions:**

The newly developed LFA allows rapid and reliable detection of ASFV, at field and laboratory level, providing a new useful tool for control programs and in situations where laboratory support and skilled personnel are limited.

## Background

African swine fever virus (ASFV) is a large, enveloped, icosahedral double-stranded DNA virus that belongs to the *Asfaviridae* family, genus *Asfivirus* [1]. ASFV was first identified in 1921 in Kenya as the cause of lethal hemorrhagic disease in domestic pigs [2]. In Europe, ASF was introduced to Portugal in 1957, and from 1960, in other countries such as Spain or Italy and the Caribbean islands, but finally eradicated. Currently, the disease is endemic in the majority of Sub-Saharan countries and Sardinia (Italy) [3, 4]. Since the introduction of ASFV into Georgia in 2007 from East Africa, several cases have been declared in Armenia, Azerbaijan and in the Russian Federation, where continued uncontrolled spreading poses a serious threat to the swine industry worldwide [5–8]. The disease again manifested itself in early 2014, when the first cases of ASF in wild boar in Lithuania and Poland were reported in areas bordering on Belarus. Since then, the ASFV has spread in Estonia, Latvia, Lithuania and Poland, mostly affecting wild boar and to a lesser extent domestic pigs [9, 10]. Presently, the disease is threatening other regions in Europe and Asia due to the potential persistent spillover of the virus to adjacent areas.

The natural hosts of ASFV are the domestic and wild suids, and soft ticks of the genus Ornithodoros. The infection of warthogs and bushpigs in Africa results in mild disease, often asymptomatic, with low viraemia titers, developing into a persistent infection in most cases [4, 11–13]. These animals act as reservoir hosts of ASFV in Africa. On the other hand, infection of domestic pigs, European wild boar, and American wild pigs leads to more acute diseases with high rates of morbidity and mortality [14].

At present, no treatment or vaccine is available to prevent ASF and the control strategy mainly relies on enforcement of sanitary measures and slaughtering of infected and exposed animals [15]. Therefore, early and specific diagnosis of the infection is urgently required for prevention, control, and eradication of the disease in affected countries. In the majority of cases, the severe nature of the epidemic disease affecting the Eastern European countries leads to mortality with high levels of viral presence in tissues and blood [16–19]. Therefore, the ASF diagnosis in the National Reference Laboratories of the affected countries should always include the detection of the virus, the antigen or the ASFV genome. The OIE-recommended tests for virus detection include virus isolation, fluorescent antibody test and both real-time and conventional PCR assays [20, 21]. However, these methods are still rather time consuming and require well-equipped laboratories and personnel, delaying the disease diagnosis in remote areas [22–25].

In the present report we describe a novel approach for detection of viral antigen by an immunochromatographic test, based on the use of a monoclonal antibody against the major viral capsid protein of ASFV, VP72. The test was first set up using semi-purified viral protein and inactivated tissue culture virus. Further studies for validation of the test were carried out using experimental and field serum samples. This novel pen-side test offers a rapid and simple assay that will allow early detection of ASFV infection, especially useful for testing wild suids in the field, and for use in small laboratories, where laboratory equipment is very simple or absent.

## Methods

### Monoclonal antibodies and sera

Monoclonal antibody (mAb) 18BG3, specific for the VP72 protein, was produced by Ingenasa, following the previously described protocol by other authors [26].

For this study, two groups of sera were used:

*Porcine samples from ASFV experimental studies*

Pigs used for experimental studies were obtained from a local commercial farm. One hundred and fifty three EDTA-blood samples were collected at regular intervals until the end of the study, from three independent experimental infections with virulent ASFV isolates belonging to P72 genotype II, currently circulating in East Europe, and IX, which is present in East Africa. The number and type of experimental samples is summarized in Table 1. Animal experiments were conducted at the BSL3 animal facilities at INIA in accordance with EC Directive 86/609/EEC, which regulates the accommodation and care of animals used for experimental and other scientific purposes. The experiments were conducted as follows:

**Table 1:** Description of the tested experimental samples collected from animals infected with ASFV genotype II and IX viruses

Clinical form	Virulence designation	ASFV Strain	Origin (P72 genotype)	N° PIGS examined	Days Post Infection (DPI)	N of samples tested	Reference
Acute	Virulent	Ken06.Bus	Kenya (IX)	6	0–14	23	Tignon et al., 2011 [27]
Acute	Virulent	Ukr12/Zapo	Ukraine (II)	6	0–12	19	Gallardo et al., 2013
Acute	Virulent	LT14/1490	Lithuania (II)	18	0–61	111	Gallardo et al., 2015 [18, 20]
	Total samples tested					153	

Experimental infections with ASFV Ken06. Bus isolate (Kenya 2006): four Landrace x Large White pigs were inoculated intramuscularly with 10 HAD50/ml of the ASFV Ken06-Bus isolate. Two untreated pigs were maintained in contact, housed in the same box as the inoculated animals. Inoculated and in-contact animals developed acute forms of clinical disease and were slaughtered or died as a result of the infection, between 8 to 17 days post inoculation (dpi)/ days post-exposure (dpe) [27, 28].

Experimental infection with the ASFV Ukr12/Zapo isolate (Ukraine 2012): four domestic pigs were inoculated by the intramuscular route with 10 HAD50/ml of the Ukraine ASFV Ukr12/Zapo isolate. The inoculated pigs were placed in contact with two naive pigs being housed in the same box. All the pigs developed peracute to acute form of the disease and died, or were ethically slaughtered, between 4 and 10 dpi (for infected pigs) and 11–12 dpe (for in-contact pigs) [29].

Experimental infections with ASFV LT14/1490 isolate (Lithuania 2014): ten naive pigs were placed in contact with eight pigs experimentally inoculated by the intramuscular route with 10 HAD50/ml of the Lithuanian LT14/1490 strain. The Lithuanian ASFV strain induced an acute disease which resulted in 94,5 % mortality. Seven of the eight inoculated animals, died or were euthanized due to the severity of symptoms between 7 and 9 dpi. One inoculated pig showed a delayed course of the disease, resembling the course observed in the in-contact animals, which died or were slaughtered between 14 and 22 dpe. One in-contact pig remained asymptomatic throughout the experiment and was slaughtered at day 61 [30].

*Porcine samples from field ASFV-infected areas within the EU*A.

Panel of 58 field EDTA-blood samples collected during the 2014 and 2015 outbreaks in EU countries (Lithuania, Poland and Estonia) were used in this study. These samples were taken from 46 wild boar and 12 domestic pigs during the surveillance programs and were sent to INIA-CISA’ by the National Reference Laboratories (NRLs) of Lithuania, Poland and Estonia for confirmatory diagnosis in accordance with the duties of INIA-CISA as EURL for ASF.

### Development and assembly of the lateral flow device

#### Capture reagents

MAb to VP72 protein (18BG3) was used as the test line capture reagent. The 18BG3 MAb was diluted to 1 mg/ml in Tris/HCl 20 mM buffer at pH 7.5 containing 5 % sucrose and 0.095 % sodium azide as preservative. The anti-control protein IgG monoclonal antibody was used as the control line capture reagent. It was diluted to 1 mg/ml in the same buffer used for the test lines. The control line is essential in order to validate the test.

The test and control capture reagents were dispensed in two parallel lines on 25 × 300 mm HiFlow Plus nitrocellulose membrane (HF120, Millipore) at 1 μl/cm. After drying for 5 min at 45 °C, the membranes were sealed and stored at room temperature under dry conditions.

#### Preparation of latex microparticle conjugates

The detector reagents consisted of 415 nm coloured carboxyl-modified latex microspheres (Estapor® Microspheres). The MAb against VP72 protein was covalently conjugated to black latex beads while blue latex particles were covalently conjugated with the LFA control detector reagent. Previously to protein conjugation, latex particles were activated with EDC (1-ethyl-3-(3-dimethylaminopropyl) carbodiimide hydrochloride) and NHS (N-Hydroxysuccinimide) and then coupled to the monoclonal antibody at a surface concentration of 1 mg/m^2^. Finally, conjugated latex particles were diluted in Tris/HCl 10 mM pH 8.2 and the microsphere solutions were stored at 4 °C before use.

#### Preparation of the conjugate pad

To prepare the conjugate solution, the MAb-latex and control-latex particles previously prepared were diluted at a concentration of 0.2 % each, in a Tris/HCl 25 mM pH 9.5 buffer containing humidity preservatives and blocking agents. The mixture was dispensed onto the rayon conjugate pad. The pads were dried for 30 min at 45 °C and stored at room temperature under dry conditions.

#### Preparation of chromatographic strips

A master card was assembled as follows: nitrocellulose membrane, conjugated pad, sample pad and absorbent pad were pasted on a plastic backing with adhesive and covered with a protector film. The master card was then cut into strips of 4.2 mm width, which were placed individually in a plastic device. A special sample pad to retain the erythrocytes (Cytosep 1662) was used, since the test was designed to be used with blood samples.

#### Test procedure

The test has been designed for use with blood samples. Blood samples must be fresh or refrigerated up to 4 days at 2–8 °C and collected with an anticoagulant (EDTA, heparin, etc.). Blood samples without anticoagulant could contain micro-clots that could block the device and result in nonspecific reactions. It is also very important to ensure the devices are stored in dry conditions, to avoid any negative effect on the result. Finally, several trials in the lab have demonstrated that the test runs correctly in a range of temperatures from 15 to 37 °C (data not shown).

Twenty microliters of sample (blood) are applied to the sample pad followed by 120 μl of running buffer (Tris/HCl pH 7.5, NaCl, casein, Tween-20 and NaN_3_ as preservative). The mixture migrates through the conjugate pad and the nitrocellulose membrane by capillarity. Results are interpreted 10 minutes after adding the sample. In the presence of ASFV, the VP72 protein is captured first by the MAb-coated microparticles, forming a latex-antibody-antigen immune complex. This immune complex then reacts with the immobilized MAb on the membrane, making the black test line visible along with the blue control line. In the case of a negative test, only a blue line appears. The blue control line must appear always; otherwise, the test has to be considered invalid.

### ASF virus detection

#### ASFV genome detection by PCR

Field and experimental samples were tested for ASFV genome detection using the OIE real-time PCR [21, 31] and the Universal Probe Library (UPL) real-time PCR [32]. The DNA was extracted from each blood sample using the High Pure PCR Template Preparation Kit (Roche Diagnostics GmbH, Roche Applied Science, Mannheim, Germany). For amplification of the ASFV genomic DNA the PCRs were carried out using undiluted and 1/10 diluted extracted DNA for each sample.

#### ASFV antigen detection by ELISA

Experimental and blood samples were tested for ASF antigen detection using a commercially available antigen detection ELISA, the Double Antibody Sandwich (DAS) ELISA test manufactured by INGENASA (®INGENASA-INgezim PPA DAS K2, INGENASA, Madrid, Spain). The samples were analysed undiluted and at 1/10 dilution according to the manufacturer’s instructions and the results obtained were compared to those obtained using the PCR assays and the new LFA developed.

#### ASF virus isolation and titration

Virus isolation was assessed in PCR-positive experimental samples using a hemadsorption assay on PBMC (Peripheral blood mononuclear cells), as described in the Manual of Diagnostic Tests and Vaccines for Terrestrial Animals [21]. The plates were examined for hemadsorption over a period of 6 days. Samples were blind passaged three times. Titres were estimated using a hemadsorption assay, to monitor the end-point dilution of ASFV isolates on PBMC by the Reed and Muench method, and expressed as 50 % hemadsorbing doses per ml (HAD50/ml) per sample.

### Statistical analysis

The concordance between each test was the overall percentage agreement between the results of the two assays calculated using two-by-two contingency tables. Kappa Coefficient (k) statistics were used to evaluate the significance of the level of concordance between results beyond that expected by chance, with k values of 0.81–1.00 representing almost perfect agreement, values of 0.61–0.80 substantial agreement, values of 0.41–0.60 good agreement, values of 0.21–0.40 moderate agreement, values of 0.01–0.20 slight agreement, and values of 0.00 no agreement (48). From the overall analysis of the results the final sensitivity and specificity of the new assay were calculated using the results of the UPL-PCR as a reference test for virus detection. Values measured included the number of true positives (TP), the number of true negatives (TN), the number of false positives (FP) and the number of false negatives (FN). Sensitivity was calculated as 100 × TP/(TP + FN), specificity was calculated as 100 × TN/(TN + FP).

## Results

### Development of the immunochromatographic assay

A monoclonal antibody, 18BG3, against the VP72 protein of ASFV, was used as the capture reagent on the test line in the LFA for ASFV detection. A specific monoclonal antibody for the control protein was used as the control line.

**Fig. 1:**
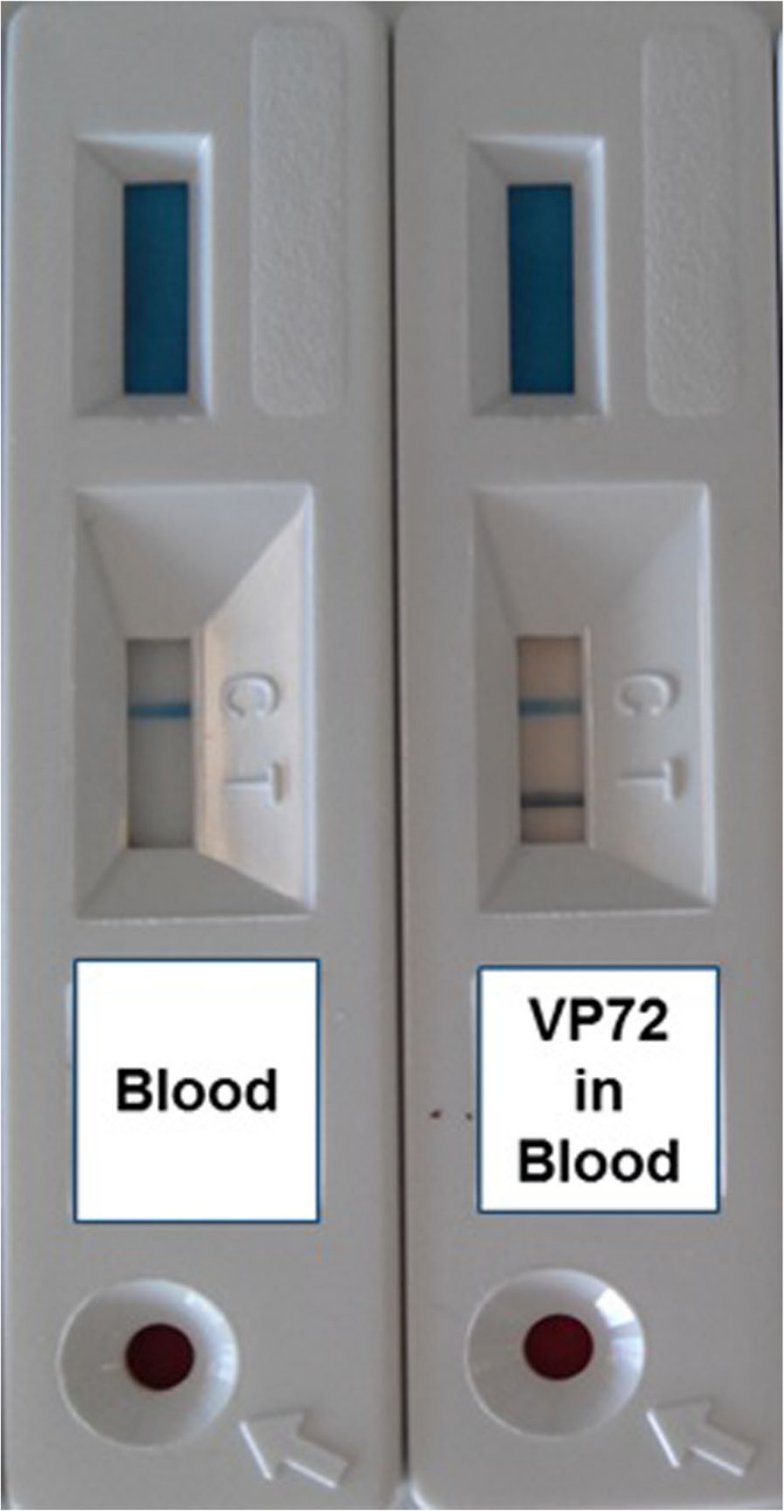
Picture of the lateral flow device for ASFV detection. The left device shows a negative result using a blood sample from a healthy donor pig; where only the blue control line is detected. The right test shows a positive result by spiking the semi-purified VP72 protein in blood; in this scenario, the two lines, control line (*blue*) and test line (*black*) will appear

Since it is known that over 90 % of the virus is associated with red blood cells [33], our purpose was to adapt the test to be used not only with serum samples, but more importantly with blood samples. Therefore, a special sample pad was used in the design of the test, in order to retain the erythrocytes, allowing the free virus to move along the strip. Besides, different running buffers containing detergent were tested in order to release as much virus as possible from the erythrocytes. Different colored beads were analyzed for antibody conjugation, taking into consideration that in case of hemolized blood samples, hemoglobin would move along the strip, staining it and thus making the visualization of the test line more difficult. For this reason, black latex beads were chosen to develop the test. Moreover, different antibody concentrations for conjugation to the latex beads were also studied to determine optimal conditions. Finally, the test protocol was established as follows: after addition of the sample to the round window of the device, the running buffer is added and after ten minutes, the results must be interpreted. Appearance of a black test line and the blue control line indicates a positive result, while appearance of the control line alone indicates a negative sample (Fig. 1).

### Performance of the LFA for ASFV detection

**Fig. 2:**
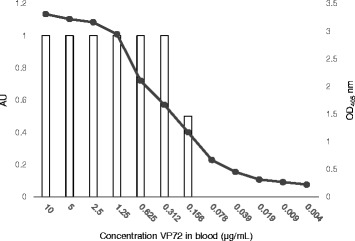
Comparison of the performance of the LFA and the commercial DAS-ELISA. Serial dilutions of semi-purified VP72 protein in blood were tested by LFA and Double antibody sandwich ELISA (DAS-ELISA) and results were compared. Both assays are based on MAbs specific for VP72 protein. The detection limit of both tests was very similar (3 ng viral protein/test by LFA *vs* 1,9 ng viral protein/test in ELISA)

In order to test the performance of the newly developed test, first experiments were carried out using semi-purified VP72 viral protein and inactivated tissue culture virus, BA71 strain. A spike-in test was assessed in the laboratory by adding the viral semi-purified protein to blood from a healthy donor pig used as the matrix. Serial dilutions of the viral protein in blood were analyzed by LFA. The analytical sensitivity of the assay was down to 3 ng/test, comparable to that of a commercially available double antibody sandwich ELISA (INgezim® PPA DAS) with 1.9 ng/test (Fig. 2). When the test was performed using the tissue culture virus, the detection limit was down to 10^4^ pfu/ml (data not shown).

### Evaluation of the LFA for ASFV detection

Out of the 153 blood samples collected at different times post infection from the 30 experimentally infected pigs, the number of positives using the UPL rt-PCR was 60 (39.21 %), 57 (37.25 %) using the OIE rt-PCR and 41 (26.79 %) using the LFA. Thirteen out of the 19 samples (68.42 %) which gave a false negative result with the LFA were correlated to blood samples with rt-PCR Cycle Threshold (C_T_) values above 30, mainly collected at initial stages of the infection or from in-contact animals exposed to the virulent Lithuania 2014 ASFV isolate (LT14/1490), which remained asymptomatic throughout the experimental infection (Table 2). When comparing with the virus isolation, the LFA was unable to detect the presence of ASFV antigen in blood samples with viral loads below 10^4^ HAU. Good correlation was observed between the LFA and the antigen INgezim® PPA DAS ELISA with just 2 of the 41 ELISA positive samples testing negative in the LFA. Among the negative samples, two were tested as false positives by using the LFA whereas only one produced a false positive when tested with the antigen-ELISA.

**Table 2:** Description of the false negative (FN) samples using the LFA by the analysis of experimental samples collected from animals infected with ASFV genotype II and IX viruses

ASFV Strain	ID PIG	Days post infection	UPL-PCR CT value	OIE real-time PCR CT value	Virus isolation (TITER _HAD50/m_l)	Ag-ELISA (INGENASA).
LT14/1490	L10	13	30.88	30.39	3,16 × 10^3^	FN*
LT14/1490	L10	17	17.61	16.49	6,81 × 10^5^	POS
LT14/1490	L11	13	26.43	27.41	3,16 × 10^2^	FN
LT14/1490	L12	13	36.67	37.21	NEGATIVE	FN
LT14/1490	L13	3	32.73	32.47	NEGATIVE	FN
LT14/1490	L14	17	33.04	34.79	NEGATIVE	FN
LT14/1490	L15	13	25.93	24.71	6,81 × 10^4^	FN
LT14/1490	L15	17	18.27	16.73	6,81E × 10^6^	FN
LT14/1490	L16	13	32.41	32.62	NEGATIVE	FN
LT14/1490	L17	3	37.46	37.45	NEGATIVE	FN
LT14/1490	L17	7	23.14	23.07	3,16 × 10^5^	POS
LT14/1490	L18	17	36.95	FN	NEGATIVE	FN
LT14/1490	L18	34	38.51	FN	NEGATIVE	FN
LT14/1490	L18	38	38.46	FN	NEGATIVE	FN
LT14/1490	L2	14	34.54	33.95	3,16E + 01	FN
LT14/1490	L3	3	32	33.04	NEGATIVE	FN
LT14/1490	L4	10	31.67	31.85	3,16E + 03	FN
LT14/1490	L6	14	27.2	26.91	3,16E + 08	FN
Ken06.Bus	CC6	D10	35.34	39.63	NEGATIVE	FN

*false negative

To evaluate the ability of the new LFA to diagnose ASF during the current epidemic outbreaks in the EU countries, 58 samples collected in 2014 and 2015, from domestic pigs (12) and wild boar (46) from affected EU countries were included in the study. The samples were tested in parallel using the two real time PCRs, the antigen-ELISA and the LFA. As with the results obtained using the experimental blood samples, the number of positive samples detected by the UPL-rt-PCR was 52 (89.65 %), whereas the number decreased to 51 (87.93 %) with the OIE rt-PCR, 35 (60.34 %) with the LFA and 28 (48.27 %) with the INgezim® PPA DAS ELISA assay (Table 3). Contrary to what was observed in the analysis of the experimental samples, only 7 out of the 17 (41 %) samples which resulted false negatives using the LFA displayed Ct values above 30 (Table 4).

**Table 3:** Comparison between the two PCR tests, the antigen-ELISA and the LFA used to detect ASFV in field-collected blood samples from wild boar and domestic pigs during the epidemic outbreaks in EU countries

Country	Host	UPL real-time PCR		OIE real time PCR		LFA		Ag-ELISA INGENASA	
		N° positives/total	%	N° positives/total	%	N° positives/total	%	N° positives/total	%
Estonia	Wild boar	1/1	100	1/1	100	0/1	0	0/1	0
Latvia	Wild boar	1/1	100	0/1	0	0/1	0	0/1	0
Lithuania	Wild boar	20/26	76.92	20/26	76.92	9/26	34.61	8/26	34,61
	Domestic pig	11/11	100	11/11	100	10/11	90.9	6/11	90,9
Poland	Wild boar	18/18	100	18/18	100	15/18	83.3	13/18	83,3
	Domestic pig	1/1	100	1/1	100	1/1	100	1/1	100
Total		52/58	89.65	51/58	87.93	35/58	60.34	28/58	48.27

**Table 4:**
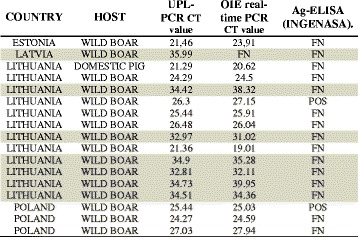
Description of the false negative (FN) samples using the LFA by the analysis of field samples

In grey are labelled false negative samples with Ct > 30 by using the UPL-real time PCR

From the results obtained in both experimental and field samples collected from animals with known infectious status and selecting the UPL-PCR as the reference method, the sensitivity and specificity of the LFA were calculated. Of the 112 ASF positive samples, there were 36 false negatives from the LFA, resulting in a sensitivity value of 67.86 %. The number of false positive samples was 2 out of 99 giving a specificity of 97.98 %. When comparing the LFA results with those obtained using the INgezim® PPA DAS ELISA assay, the Kappa values of 0.92 [CI 95 % = 0.86–0,98] showed almost perfect agreement between the two assays.

## Discussion

Early diagnosis of African swine fever is essential in order to establish effective surveillance programmes and control measures to prevent the spread of ASF. Rapid, sensitive and specific assays are required by diagnostic laboratories. A positive diagnosis involves the detection and identification of ASFV-antigens, antibodies or DNA in a given sample. The development of new easy-to-handle diagnostic tools designed for non-specialized front-line laboratories with limited equipment and allowing deployment at field level, would be very helpful in improving ASF control programmes. This applies particularly to positive results, allowing for control measures, such as restriction of animal movement, to be adopted immediately, until confirmation from official reference laboratories is received. In case of virulent strains, animals usually develop high levels of viremia, dying soon after infection. Virus, antigen or genome detection is very useful to diagnose these hyperacute/acute forms of the disease. In case of ASFV infection with moderate or low virulent strains, these usually induce high or medium viremia levels at the beginning of the infection, and high antibody titers from the second week post infection. In these cases, both virus and antibody detection must be used to obtaint a reliable diagnosis.

In the present study, a lateral flow assay for antigen detection was developed and tested with blood samples from pigs inoculated experimentally with ASFV and from field infected animals. The assay was based on a monoclonal antibody to the VP72 protein, which is the major capsid protein of ASFV [34, 35] and therefore an appropriate target antigen for virus detection. The data obtained by the LFA were compared with those obtained by both rt-PCR (UPL rt-PCR and OIE rt-PCR) and by INgezim PPA DAS. It has been recently described that the UPL-rt-PCR is the most sensitive and trustworthy method for detecting ASF, followed by the OIE-rt-PCR [20]. In that study the INgezim PPA DAS was included for comparison purposes, showing a good-to-moderate agreement with the UPL-rt-PCR (k = 0.67 [95 % CI, 0.58 to 0.76]). The data analyzed in the present work showed that the sensitivity of the LFA was lower than that obtained by either rt-PCR, as was expected, detecting 68 and 72 % of the positive samples from experimentally infected animals, in comparison with UPL-rt-PCR and OIE-rt-PCR, respectively. Besides, more than 50 % of the false negative results by LFA corresponded to samples with C_T_ values higher than 30, obtained from second week of infection and onwards, and therefore with low viral loads. Nevertheless, when compared to the results of the antigen-ELISA, only two false negative samples were detected by LFA. It is important to take into account that these two techniques are detecting viral antigen, while the rt-PCRs detect viral genome, and therefore being more sensitive. In any case, the specificity of the newly developed test was very close to 100 %, indicating a low percentage of false positive results, which is crucial in order to avoid unnecessary states of alarm and consequent expenses.

In a similar way, when the field samples were analyzed by the LFA, 67 % of positivity was found in comparison with the reference technique, UPL-rt-PCR. However, in this case, the majority of the false negative results did not show Ct values above 30. This discrepancy could be due to the quality of the sample, since these field samples came mostly from carcasses of animals found dead or hunted. This aspect is critical in the analysis of the samples by LFA, affecting the result even if the viral loads are high enough to be detected by this method.

Although PCR is the technique most frequently used for viral detection, it is a laborious technique, which requires good training to avoid contamination and therefore false positive results. Besides, samples need to be transported quickly to the corresponding laboratory in order to obtain a result. Although LFA is less sensitive than rt-PCR tests, this novel pen-side test offers some advantages and benefits, especially in field scenarios: it is a rapid, economic and simple-to-use diagnostic tool, since it does not require any kind of instrumentation and the result is interpreted at a glance and with very high specificity. Furthermore, the test has been designed to be used with blood, thus making sample processing easy and feasible. All these features make these devices very suitable for field application or basic laboratories, especially in countries where laboratory infrastructure is deficient or even absent. Further validation is currently ongoing in different countries in Europe, Asia and Africa. Field validation is particularly crucial before these tests can be used as front-line diagnostic assays. This should include samples not only from domestic pigs, but also wild boar.

## Conclusion

In conclusion, the newly developed LFA allows the detection of ASFV with viral loads from 10^4^ HAU, corresponding to days 4–7 depending on the virulence of the viral strain and depending on the condition of the sample. This is a very valuable tool accompanying the antibody detection tests (including pen-side tests) in any scenario. It will assist especially local veterinary services, where in many cases first evidence of the disease is based only on clinical symptoms.
